# Automated particle analysis of D.B. Cooper's tie

**DOI:** 10.1111/1556-4029.70372

**Published:** 2026-05-28

**Authors:** Thomas G. Kaye, Kent L. Rhodes

**Affiliations:** ^1^ Foundation for Scientific Advancement Sierra Vista Arizona USA; ^2^ McCrone Group Westmont Illinois USA

**Keywords:** automated particle analysis, cold case investigation, D.B. Cooper, energy‐dispersive x‐ray spectroscopy (EDS), scanning electron microscopy (SEM), SEM‐EDS, trace evidence

## Abstract

Automated particle analysis (APA) using scanning electron microscopy (SEM) and energy‐dispersive x‐ray spectrometry (EDS) is a well‐known method in forensics, especially as it is applied to gunshot residue analysis. Here we describe the analysis of particles lifted from D.B. Cooper's tie, which was left on the hijacked plane in 1971. A Tescan MIRA4 SEM was used to locate, image, and analyze ~180,000 particles on a single 12‐mm SEM stub used to sample the Cooper tie. Initial analysis of the data for unusual or distinctive elemental compositions found many interesting particles, including titanium–antimony, silver–mercury, gold–palladium, and uranium. Due to the short acquisition times in APA, elemental detection is limited to major and some minor elements. Therefore, it is crucial to relocate particles of interest for deeper analysis by SEM‐EDS. Particle identifications can be confirmed or revised after consideration of longer count time EDS spectra and more detailed SEM imaging. The types and quantities of particles found on the Cooper tie suggest specialty metals (titanium, gold–palladium, mercury–silver, tungsten–cobalt) and industrial facilities (industrial aluminosilicate minerals) as possible associations. Although some previously identified materials of interest were not supported by the 2024 reanalysis results, other new targets of interest were identified. In particular, the mineral particles on the tie may offer a fresh look into Cooper's geographic locality and activities. This work also highlights the forensic value of a tie as an unwashed particle reservoir.

**Highlights**

- Ties can preserve environmental particulate evidence relevant to forensic history.
- Automated particle analysis identified >180,000 particles from one necktie.
- Confirmatory SEM‐EDS ruled out suspected Ti–Sb alloy particles.
- Particles on the Cooper tie suggest links to specialty metals and industrial facilities.
- Automated trace particle analysis offers new insight into cold‐case evidence.

## 1 INTRODUCTION

**FIGURE 1:**
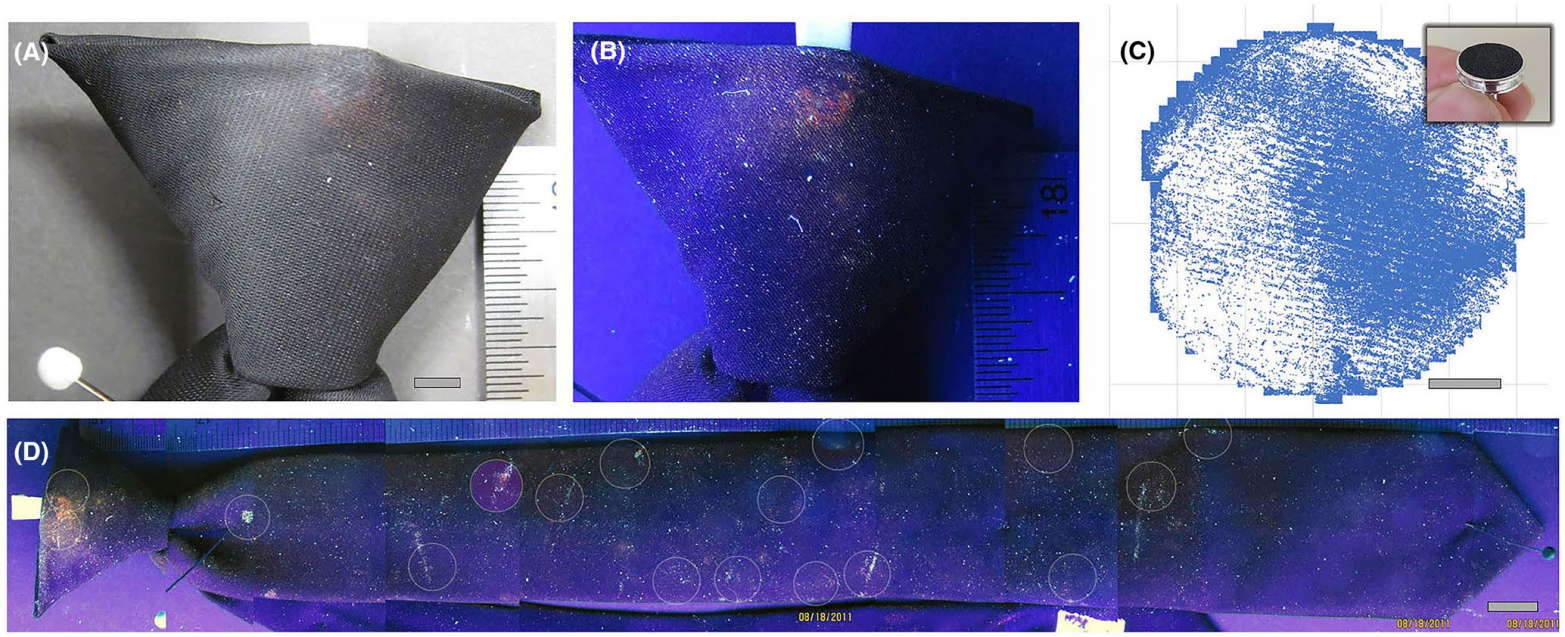
(A) Knot from D.B. Cooper's clip‐on tie, white light. Scale bar 10 mm. (B) Tie knot under long‐wavelength ultraviolet light showing a high concentration of particles. (C) Particle distribution map for stub #675 showing locations of ~180,000 particles. Scale bar 2 mm. C inset, 12‐mm stub used in the investigation. (D) Tie under long‐wavelength UV showing the abundance of particles on the tie as well as strategic sampling points for the SEM stubs. Both the front and back of the tie were sampled. Scale bar 15 mm.

The 1971 hijacking of a Boeing 727 out of Portland by the now‐famous D.B. Cooper remains the only unsolved hijacking in United States history [1]. Cooper mistakenly left his clip‐on tie on the seat, and it remains today as one of two important pieces of hard evidence from the case (Figure 1D), the other being a portion of the money found 10 years later on the Columbia River north of Portland [2]. Ties are typically never washed, and this offers a repository for environmental particles to accumulate everywhere the wearer has been. In 1971, the scanning electron microscope (SEM) was in its infancy, and it was not widely used in forensics until the mid‐1970s [3, 4]. A modern‐day SEM investigation [5] of the tie particles holds the possibility of revealing new insights into the criminal's occupation or geographic location [6, 7].

In 2008, the tie was sampled for surface particulate. A manual investigation of ~700 particles was carried out by randomly locating and elementally analyzing larger particles of interest using SEM with energy dispersive x‐ray spectroscopy (EDS). In 2016, an automated SEM–EDS analysis was performed that produced a ~110,000 particle dataset aggregated from analyzing 10 different SEM stubs collected from the tie. There were difficulties in relocating the particles even with landmarks, perhaps due to mechanical issues with the stage accuracy. In 2024, it was decided to re‐analyze one stub that held particularly interesting particles using improved automated analysis methods.

## 2 MATERIALS AND METHODS

**FIGURE 2:**
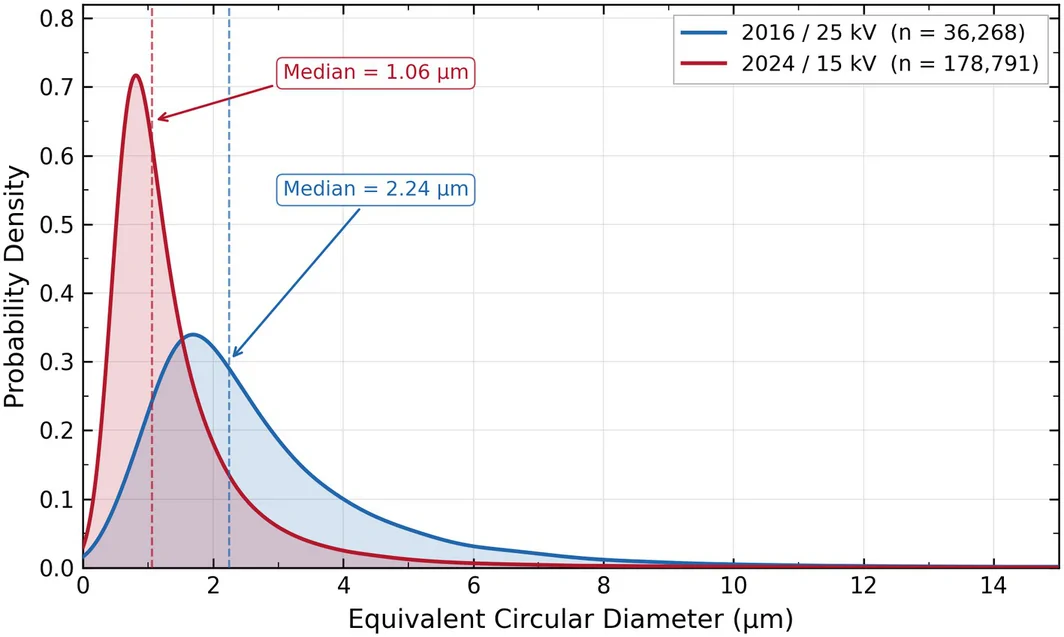
Particle size distributions from 15 kV (2024 analysis) and 25 kV (2016 analysis) using APA. Analysis at 15 kV improves the detection of very small particles (<1 μm).

The reanalysis was performed using parameters to maximize the detection of small particles of a wide range of materials. This included a lower accelerating voltage of 15 kilovolts (kV) for better spatial resolution and a smaller interaction volume, and larger area EDS detectors. Improvements in the SEM instrument and computer systems also improved particle throughput. The surprising result was ~180,000 particles detected on this single stub. As noted in the particle map of Figure 1C, the distribution was so dense that one can see the density and orientation of the tie's fabric weave. Figure 2 shows that analysis at 15 kV improves the detection of very small particles (<1 μm). The 2024 reanalysis provided a large dataset for study in order to try to constrain the environment in which D.B. Cooper may have worn the tie [8] and offered an opportunity to add new information to the case.

### 2.1 Sampling

Sampling in 2008 used 12‐mm SEM stubs (Figure 1C inset) covered with carbon sticky tabs to collect particles from various places around the tie. Stubs were curated in dedicated stub boxes to prevent contamination. This first batch of sample stubs was designated Group #1. Subsequently, after manual examination of the Group #1 stubs by SEM and realizing the quantity of particles on the tie, a second round of sampling of the tie was performed in 2011, again using carbon sticky tabs on SEM stubs. This batch of sample stubs is identified as Group #2. Care was taken during the second sampling to record the precise positions of the sampling locations (Figure 1D).

Prior to the Group #2 sampling, a standard long‐wavelength ultraviolet (UV) lamp was used to image the tie, and this revealed the distribution of fluorescent particles (Figure 1D). The UV image revealed an especially dense area of particles at the tie knot finger push point. Although not all particles of interest will fluoresce, the presence of a large amount of fluorescent particulate at the tie knot made it a prime target of the Group #2 sampling (Figure 1B).

### 2.2 SEM‐EDS analysis

All stubs were coated with gold or palladium using a Bio‐Rad E5000 M coater prior to SEM analysis. The Group #1 stubs were manually analyzed shortly after collection with a JEOL T300 SEM and a Kevex EDS system. Approximately 700 particles were randomly selected for SEM‐EDS analysis.

In 2016, the combined Group #1 and Group #2 sets of stubs were automatically analyzed by The McCrone Group using their ASPEX Explorer SEM, which provided a total of approximately 110,000 particle EDS spectra and thumbnail SEM images from 10 different SEM stubs collected from the tie using a 0.25‐s collection time at 25 kV.

### 2.3 2024 Reanalysis

In 2024, a single stub #675 from the center of the tie knot was rescanned with The McCrone Group's TESCAN MIRA4 thermal field emission scanning electron microscope using the combined energy dispersive x‐ray signal from four EDAX Element‐V silicon drift detectors. The entire surface of the stub was analyzed using a 15 kV/500 picoampere (pA) electron beam. Automated analysis was performed using the SEMantics extension to NIST DTSA‐II software for instrument control and data acquisition [9]. Using a 0.4‐s acquisition time, the throughput rate was >6000 particles per hour. Individual thumbnail SEM images and EDS spectra were collected for each particle. The completed scan took 29 h.

The particle data were analyzed using NIST Graf software [10]. The EDS spectra were quantified using multiple linear least squares (MLLS) fitting to commercial, elemental and compound standards measured using a 15 kV/500 pA electron beam [11]. Standard spectra are collected so that the counting statistics are much better than the unknown particle spectra, typically for a live time of 60/*C* seconds, where *C* is the weight fraction of the standard element. Although a full matrix correction for quantitative analysis was not performed on the full dataset, the Graf software produces normalized k‐ratios that are corrected for electron dose and concentration of the element in the standard.

Particle classification was performed using a custom, manually developed rule set and cluster analysis. This produced a material identification for each of the particles in the dataset. Particles of interest, based on elemental composition and morphology, are present in very low numbers on the tie. The baseline abundance of these rare particle types on unwashed ties from different environments is not yet established. Further analysis of control ties from varied settings would be necessary to distinguish occupational or environmental exposure from ambient background.

**FIGURE 3:**
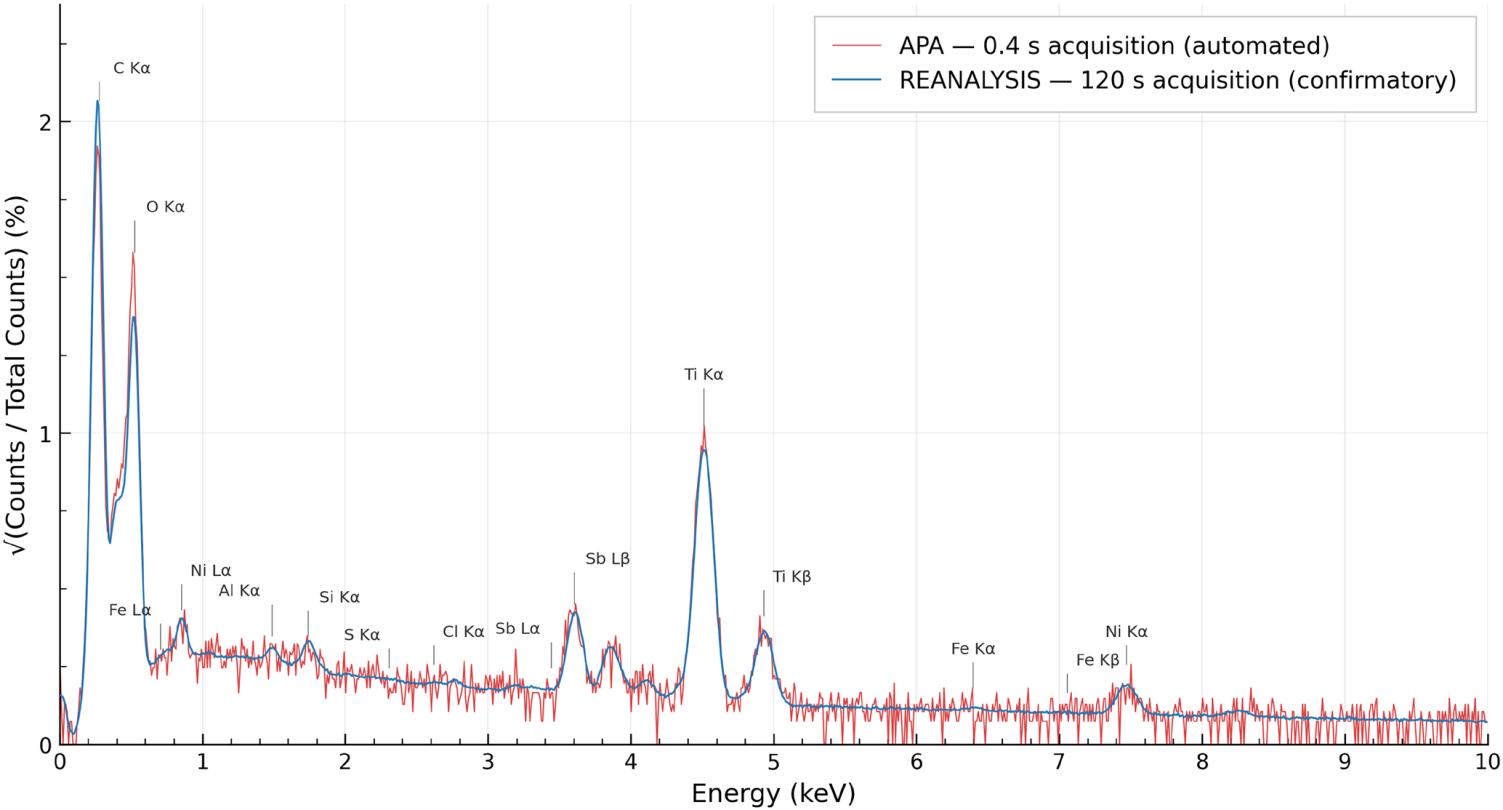
EDS spectra of a 0.5‐μm Ti–Sb particle. The RED spectrum is from automated particle analysis (APA) using a 0.4‐s collection time. The BLUE spectrum is from a 120‐s collection time reanalysis of the same particle. The spectra are scaled to the square root of the integrated counts in each spectrum. Minor and trace elements are better resolved with a longer collection time. [Correction added on 01 September 2026, after the first online publication: The figure 3 has been updated in this version.]

Follow‐up targeted particle analysis on Stub #675 was done on a Tescan Vega II SEM with EDAX Octane Pro and Team software. Exposures were typically 20 min at 20 kV, with less than 50% dead time. The extended integration time allowed for much improved signal‐to‐noise, which allowed detection of much lower concentration elements [12]. Figure 3 shows EDS spectra of a 0.5‐μm particle of Ti–Sb analyzed at 0.4 s and 120 s acquisition time. The longer, targeted analysis allows minor and trace elements to be better resolved.

## 3 RESULTS

While three independent surveys were done on the tie stubs, the 2024 analysis of Stub #675 provides the primary dataset discussed here, supplemented by relevant findings from the 2008 and 2016 campaigns. In total, 40 different elements were detected in various combinations. The majority of particles were 1 μm or smaller in size (Figure 2).

Various methods were used to identify particles of interest in the Stub #675, ~180,000 particle dataset. These methods included manual inspection, automated clustering, rule set classification, and targeting based on the previous analysis campaigns. The particle classifications were reviewed for unusual or distinctive materials, such as metal alloys, uncommon elements, and anthropogenic particle morphologies. From this review, 61 particles were selected for relocation and further deeper analysis by SEM‐EDS (See Table S1). These particles are primarily those with rare or unusual combinations of elements, but also include some particle types not previously identified as potentially of forensic interest. These 61 particles are not presented as the only potentially interesting particles in the dataset, but rather as the most obvious targets for further analysis based on this classification and review.

Notable types are particles with compositions of:

Titanium
Titanium–antimony
Gold and palladium in varying proportions
Mercury–silver
Uranium‐rich
Tungsten–cobalt
Zirconium‐rich
Antimony‐rich
Zircon mineral.

In addition to these particles, large numbers of aluminosilicate particles were found on the tie sample. These particles will also be discussed.

## 4 DISCUSSION

### 4.1 Materials of interest

#### 4.1.1 Titanium

**FIGURE 4:**
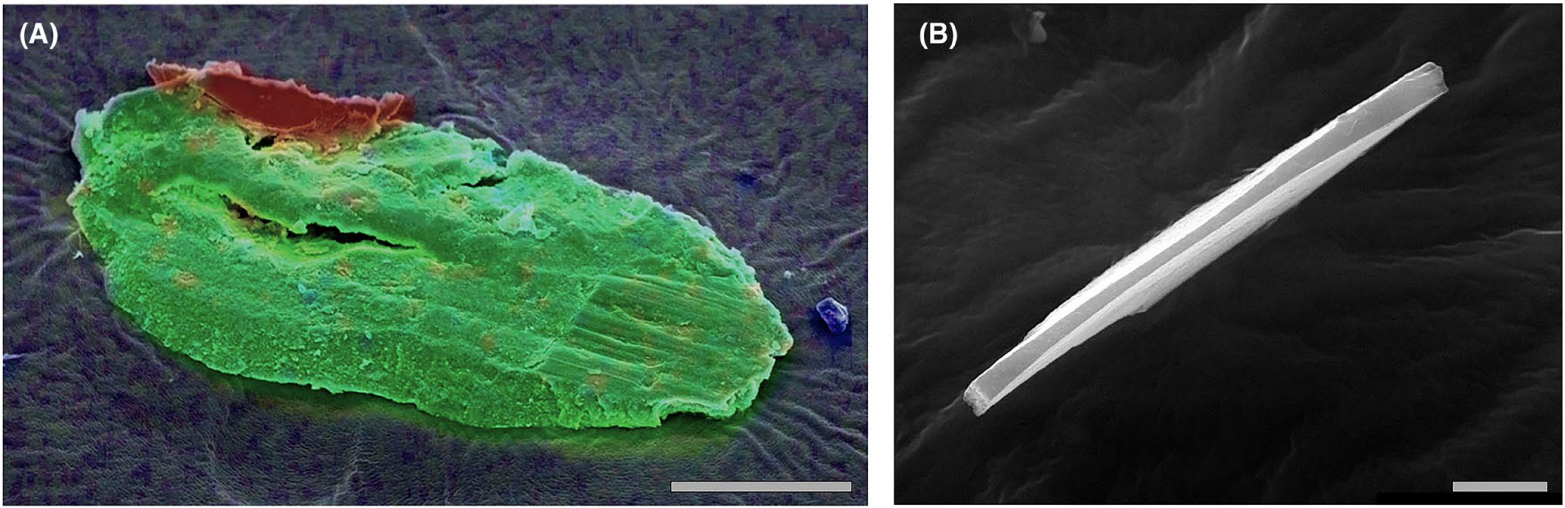
Titanium particles were identified in a 2008 manual analysis of tie samples. (A) Titanium particle (green) with embedded stainless steel (red). The stainless steel component has the appearance of being smeared into the titanium. Scale bar 100 μm. (B) Pure titanium shard found on one stub. Elemental analysis showed that both particles were not alloys. Scale bar 10 μm.

In the 2008 Group #1 analysis prior to the automated scan, two metallic shards of pure titanium metal were found (Figure 4A). In the automated scans of 2016 and 2024, no additional definitive titanium metal particles have been confirmed. Boeing was developing the all‐titanium supersonic transport (SST) during this time, and several months before Cooper jumped, the SST was canceled, and many were laid off. It had long been speculated that he worked at Boeing, and even though the SST used alloyed titanium and the tie particles were pure titanium, the particles generated a lot of speculation about him as a Boeing employee. A titanium metal flake with stainless steel smeared into it had been found in the initial Group #1 manual search and provided one of the most unique findings from that series.

Measurement of oxygen and other light elements in small particles is difficult because of the strong absorption of low‐energy x‐rays, irregular particle geometry, surface contamination, and quantification algorithms not designed for particles [13]. Often morphology and surface texture are more reliable indicators of metallic character than the measured oxygen content.

#### 4.1.2 Titanium–Antimony

Initial research into the 2016 McCrone data found three particles on the tie knot containing titanium and antimony with percentages that matched an early patent for a Ti–Sb alloy that was never produced commercially. Without rotational landmarks from the first automated run of the stubs, the particles could not be relocated for a deeper analysis. Searching for Ti–Sb particles was a primary factor in the re‐analysis of stub #675 in 2024 since it would potentially constrain the search to a single company.

**FIGURE 5:**
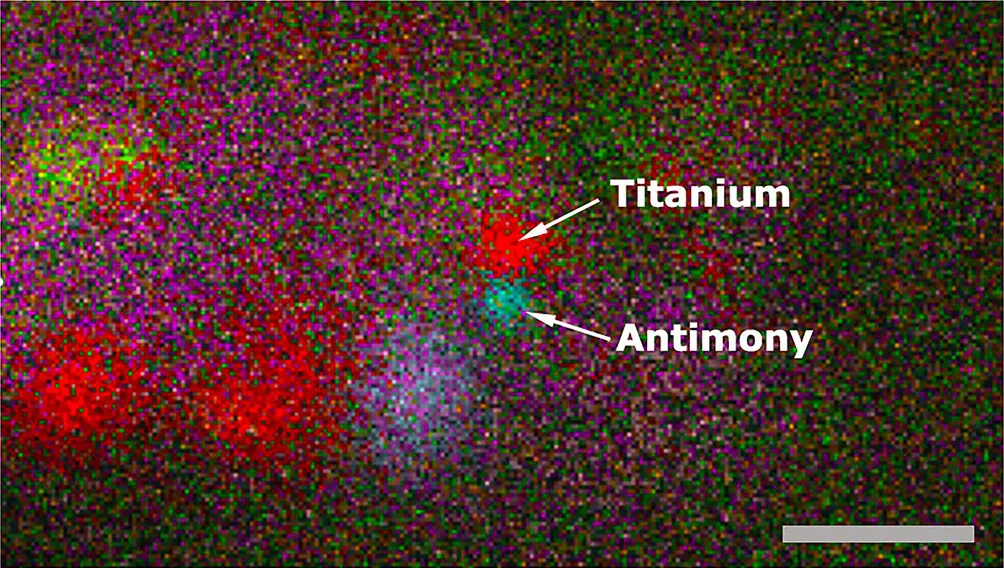
Elemental map of a titanium–antimony particle. The particle was originally thought to be an alloy because the spectrum showed both elements, but closer examination under the SEM shows this is an aggregation of particles that is not a homogeneous material. Scale bar 2 μm.

The 2024 reanalysis of stub #675 showed 17 particles containing titanium and antimony, of which 12 were relocated and analyzed. All the particles which initially looked like good candidates for a refined metal, that is, high percentages of titanium and antimony, were dismissed as non‐metallic rather than Ti–Sb alloy after deeper examination. None was found to unequivocally be metallic; one was found to be an agglomerate with separate titanium and antimony particles side by side, which could easily have been detected together in an EDS spectrum (Figure 5).

An alternative source for the Ti–Sb particles may be the common pigment titanium yellow [14], which is a good match to the composition and morphology of the tie particles. Other pigment particles were identified in the 2016 analysis of the tie particles.

Particles containing major concentrations of antimony, without titanium, were found on the tie and confirmed by relocation and deeper examination. Antimony is not a common element, especially as a pure element or compound. Antimony compounds are used in flame retardants [15].

#### 4.1.3 Gold–Palladium

Gold and palladium particles were found in the center of the tie in the first Group #1 manual investigation. They were discounted because a gold‐plated tie clip was found attached to the tie, and there were puncture marks from tie tacks, so it was assumed these particles came from the jewelry. The 2024 reanalysis of stub #675, from the tie knot, found 1600 particles that contained gold and palladium. The high number of particles suggests that jewelry abrasion is an inadequate explanation for their presence on the knot. Deep analysis of some gold–palladium particles showed additional elements, usually silicon and aluminum.

**FIGURE 6:**
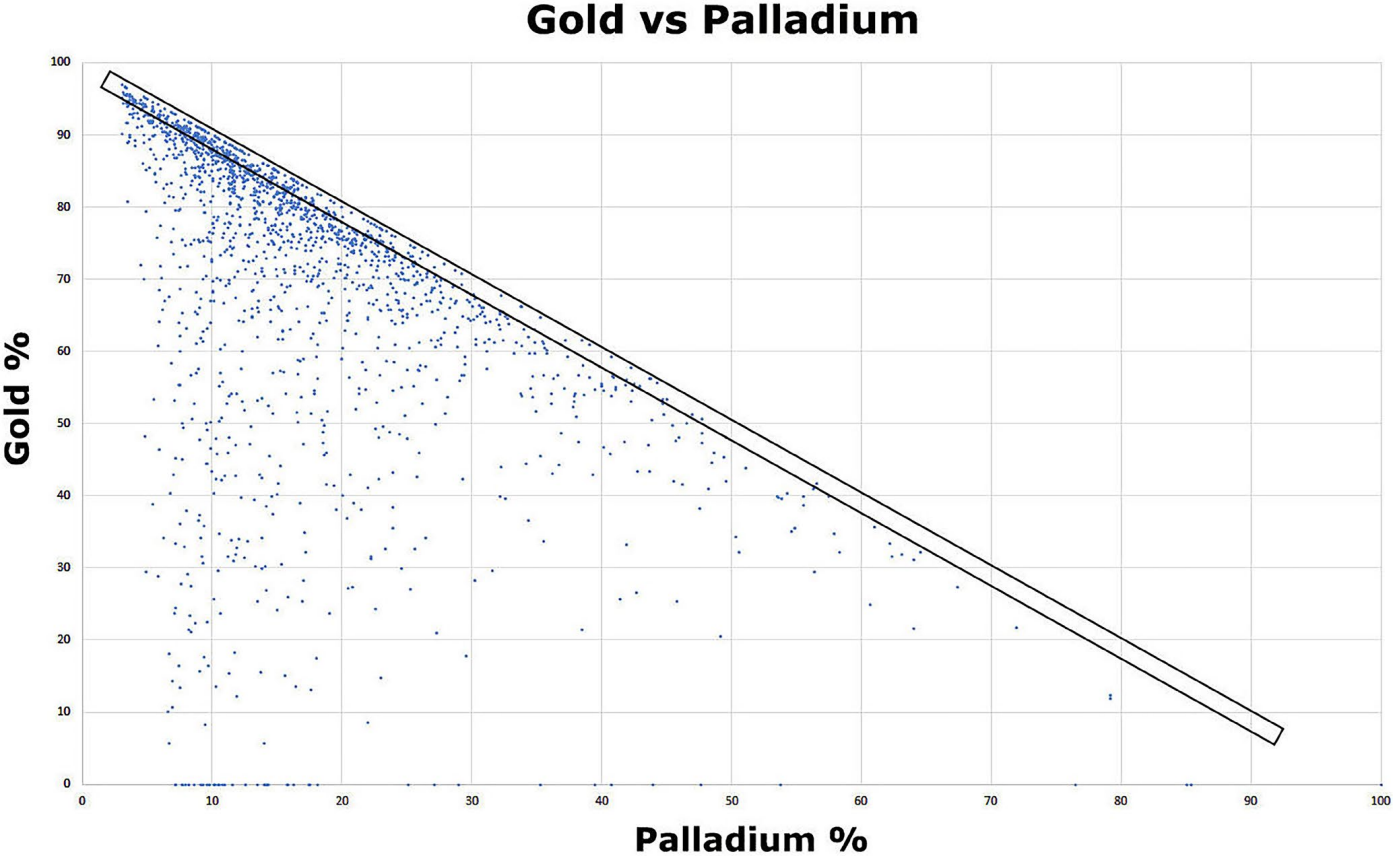
Compositional plot of the 1600 gold–palladium particles found on the tie knot. The black box encompasses a zone where the fraction of gold versus palladium varies from ~95% gold to less than ~50% gold. This discounts the idea that the particles were from jewelry since these compositions do not match common alloys.

Examination of the Au–Pd compositions of the 1600 particles (Figure 6) showed that hundreds ranged from approximately 90% Au/10% Pd to 50% Au/50% Pd, with many also containing appreciable Si and Al. Palladium is used to bleach gold in white‐gold jewelry, but reported jewelry alloys are typically multicomponent Au–Pd–Ag/Cu/Ni/Zn systems rather than simple Au–Pd particles [16]. Accordingly, the observed particles are not readily consistent with a conventional white‐gold jewelry source. Gold–palladium targets are used for metallic coating of SEM samples [17]. However, this would not explain (1) the localized nature of Au–Pd on the tie, (2) discrete particles of Au–Pd, rather than a < 20‐nm coating, and (3) the variable Au:Pd ratios in the particles.

#### 4.1.4 Mercury–Silver

Five particles of mercury–silver were detected on the knot in the 2024 stub #675 analysis. One had low oxygen at 5%, plus tin and copper, which are common constituents of amalgams used in dental fillings in 1971 [18]. However, the other particles of mercury–silver did not have tin or copper components, so this cannot be attributed to a simple dentist visit. Mercury–silver particles were not detected in the Group #1 study. Like many of the other particles, this one also had silicon and aluminum in the spectrum.

#### 4.1.5 Uranium

A single uranium‐bearing particle was found in the 2024 stub #675 analysis. A full analysis showed 70% uranium, 15% silicon, 12% aluminum, and 2% lead (excluding oxygen). The presence of lead and oxygen strongly suggests this is a natural uranium–silicon mineral or uraninite [19]. This particle prompted scanning all the stubs with a Radiacode 102 Spectral Geiger Counter/Nuclear Radiation Detector for an extended period, but no radioactivity was detected.

#### 4.1.6 Tungsten–Cobalt

The only particle examined in the 2024 stub #675 analysis that could unequivocally be identified as a metal was 95% tungsten, 4% cobalt, and extremely low oxygen. This matches cemented carbide cutting tools widely used in 1971 metal working and still in use today.

#### 4.1.7 Zirconium

Several zirconium‐bearing particles were found in the analyses. These may include zircon mineral particles (zirconium silicate). The zirconium‐bearing particles found on stub #675 are too small to undergo a detailed analytical examination for trace elements, but additional stubs from the tie may be searched for larger particles. If suitable zircon mineral particles are found, their trace element composition may be used to determine provenance if they are large enough for geochemical analysis [20].

One zirconium‐bearing particle analyzed on stub #675 was relocated for detailed analysis. This particle was too small for complete trace element analysis but was consistent with zircon with >3% hafnium. Hafnium is typically found in quantities of 1% to 2% in zircons [21]. Higher levels of hafnium suggest this particle originated in alkali granite [22]. Provenance analysis using zircons or other minerals could provide a breakthrough in tracing the Cooper tie. Automated SEM‐EDS is an excellent technique for locating suitable particles, but a conclusive provenance analysis would require additional analytical methods.

#### 4.1.8 Aluminosilicates

Aluminosilicates were found in abundance on the tie, and as some of the most common minerals [23], they were initially ignored. After reviewing the 61 re‐analyzed particles, 34 had silicon, and out of those, 14 had aluminum–silicon. This prompted a review of the entire 2024 stub #675 dataset, in which ~74,800 aluminum–silicon particles represented about ~40% of the total particle population, which seems very high considering the variety of elements found.

SEM‐EDS alone is not sufficient to identify specific aluminosilicate mineral phases in small particles, without polarized light microscopy, Raman spectroscopy, x‐ray diffraction, or other techniques. However, general patterns in SEM‐EDS data may reveal useful information about a sample. Soil particles have been used in forensic cases [24, 25] for sample comparison.

**FIGURE 7:**
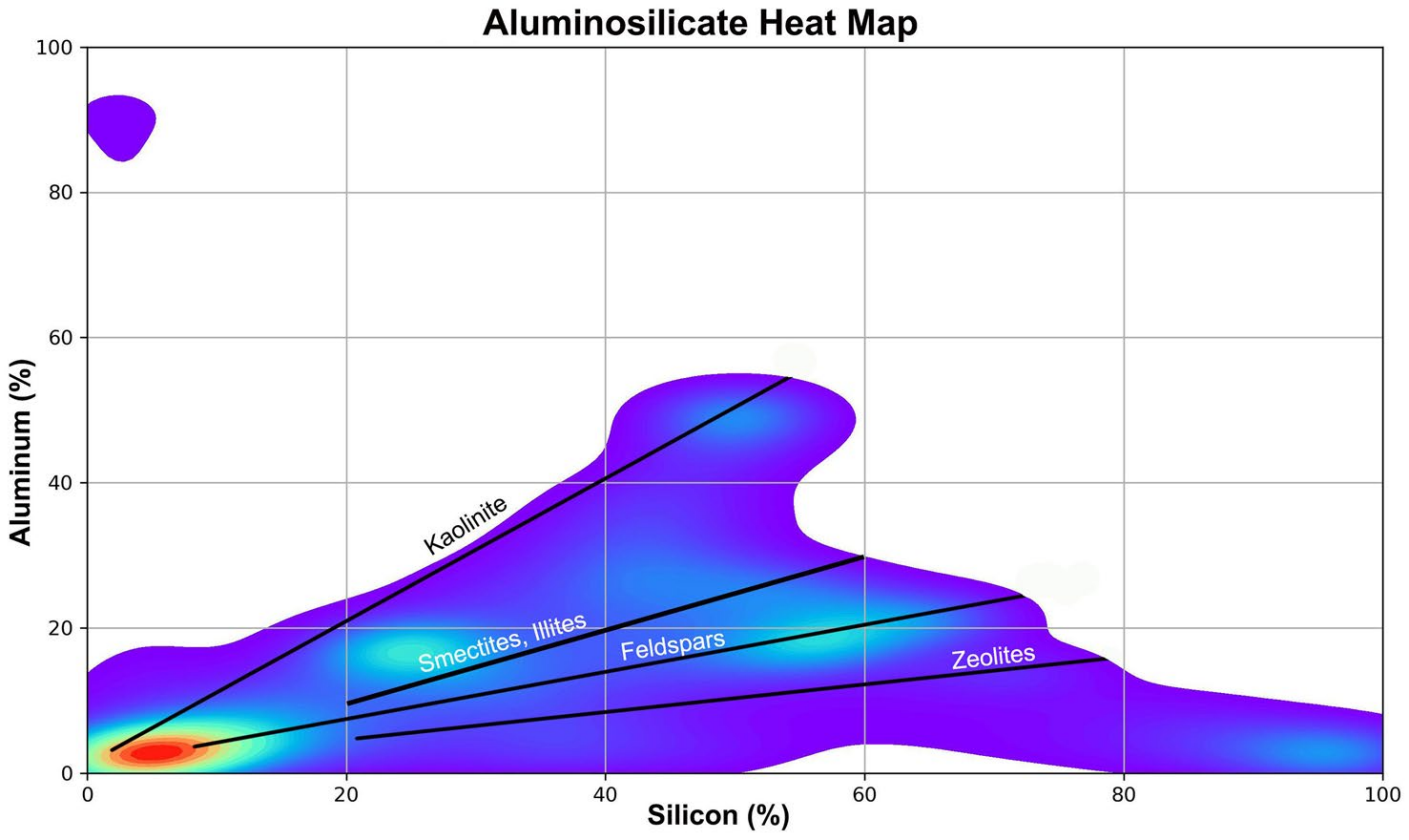
Heat map of aluminum to silicon proportions among the 74,800 aluminosilicates found on the tie. Black lines represent constant Al:Si ratios. These ratios are indicative of possible mineral groups, although SEM‐EDS alone is not sufficient to identify aluminosilicate mineral phases in small particles.

A heat map of Si and Al concentrations was generated using the entire 2024 stub #675 dataset (Figure 7). To first order, the formulas for typical common clays, rock‐forming minerals, and zeolites have the Si:Al ratios in Table 1 and are plotted in Figure 7. These ratios are indicative of possible mineral groups.

**TABLE 1:** Silicon to aluminum ratios in various aluminosilicate minerals. For the mineral groups smectites and zeolites, compositions are listed for typical examples.

Clay or mineral	Si:Al ratio
Kaolinite (clay) Al_2_Si_2_O_5_(OH)_4_	1:1
Smectite (montmorillonite) (Na,Ca)_0.33_ (Al,Mg)_2_ Si_4_ O_10_ (OH)_2_·_*n*_H_2_O	2:1
Feldspar XAlSi_3_O_8_	3:1
Zeolite (clinoptilolite) (Na,K,Ca)_2–3_ Al_3_(Al,Si)_2_Si_13_O_36_ **·**12H_2_O	5:1

The particle clusters from stub #675 show a higher abundance of particles with lower Si:Al ratios. Clays and smectites are widely used industrial minerals, whereas feldspars are the most common rock‐forming minerals. Given the differing geologic history for many aluminosilicates [26], the presence or lack of particular families as shown here may be instructive in determining a geographic locality or industrial use [27]. This evaluation of aluminosilicates on the Cooper tie is preliminary, but continued investigation of the more abundant, common particle types on the tie may help in narrowing a pool of suspects.

## 5 CONCLUSIONS

Automated particle analysis has a significant advantage over manual examination in collecting a massive quantity of data. The automated analysis data can be examined by manual inspection and by clustering methods to identify unusual or distinctive particles of interest. In addition, a sample “fingerprint” of the common particle types may allow forensic comparisons to reference samples or databases.

The speed advantage of automated particle analysis is offset by the short collection times, which mostly limit detection to major and some minor elements. Therefore, it is crucial to relocate particles of interest for deeper analysis by SEM‐EDS. Initial conclusions about elemental concentrations and particle identifications are often revised after consideration of longer count time EDS spectra and more detailed SEM imaging. Using longer integrations, modern EDS software can easily resolve most elemental overlaps and reduce ambiguity in composition.

Automated particle analysis has traditionally been used to locate a small number of distinctive particles in a large number of background environmental particles—the proverbial “needle in a haystack” problem. This is the approach for gunshot residue analysis using automated SEM‐EDS. Applied to the Cooper tie particles, this approach has identified several materials of interest (titanium metal, Ti–Sb, zircons, and others) as discussed in this paper. However, the hundreds of thousands of “environmental background” particles in the Cooper dataset may be a previously underutilized source of forensic value in tracing the wearer of the tie. The characterization of aluminosilicate minerals discussed here shows how this evidence could be used to identify areas or activities that expose the tie to these particles. Although forensic soil analysis is a well‐established method in criminal investigation [24], we suggest here that datasets from automated particle analysis may be used in a similar fashion to answer forensic questions involving locations and activities.

The 2024 automated particle analysis using a state‐of‐the‐art instrument shows the value of the method. Many more particles were found than in the analysis of 2016, primarily because of superior detection of small particles. The majority of the particles are sub‐micrometer in size. Dust transfer from fingertips while handling the tie probably deposited much of the particulate, but other deposition processes, such as environmental settling, cannot be excluded.

UV light was shown to be excellent at quickly locating particle hot spots on the tie, although not all particles of interest will fluoresce. This directly allowed determining sample sites with precision, as seen in Figure 1D.

Ties stand out as a significant potential source of environmental particles, unlike other clothing that is washed regularly. The quantity of particles found in the 2024 analysis was quite unexpected, especially considering the particles still left in the knot area were likely several orders of magnitude more numerous than the ones collected with the stub.

Modern contamination is an ever‐present possibility with these types of investigations [28, 29]. The focus here was on elements of particular rarity, not abundant in a typical office or household environment. The tie has been curated in the basement evidence storage in the Seattle office of the FBI since the 1970s. It had been sent to the FBI crime lab and several FBI offices in the years directly following the hijacking, but was only removed from the Seattle office for additional DNA testing in 2001. Presumably, the various FBI offices maintained evidence protocols with the tie.

Prior to the particle analysis, Cooper's profile was limited to a physical description and the type of cigarettes he smoked. The types and quantities of particles found on the Cooper tie suggest specialty metals (titanium, gold–palladium, mercury–silver, tungsten–cobalt) and industrial facilities (industrial aluminosilicate minerals) as possible associations. Although some previously identified materials of interest were not supported by the 2024 reanalysis results, other new targets of interest were identified. In particular, the quantity of mineral particles offers a different look into the potential geographic areas Cooper could have visited. Looking for a common denominator for all these particles is ongoing, and our entire particle dataset is included as supplemental materials so that other investigators may examine it. This is the only material evidence in the case with the potential to dramatically reduce the number of suspects.

## FUNDING INFORMATION

Partial funding for this work was provided by a private donation from Mark Meltzer.

## CONFLICT OF INTEREST STATEMENT

The authors declare no conflicts of interest.

## ETHICAL APPROVAL

This study did not involve human participants or animals. This work was presented at The DB Cooper Conference (an informal meeting), November 2024, in Seattle, WA.

## Supporting information


Table S1

Table S2

## Data Availability

The data that supports the findings of this study are available in the supplementary material of this article.
